# Global Healthspan-Lifespan Gaps Among 183 World Health Organization Member States

**DOI:** 10.1001/jamanetworkopen.2024.50241

**Published:** 2024-12-11

**Authors:** Armin Garmany, Andre Terzic

**Affiliations:** 1 Mayo Clinic Alix School of Medicine, Mayo Clinic Graduate School of Biomedical Sciences, Mayo Clinic, Rochester, Minnesota; 2 Marriott Heart Disease Research Program, Department of Cardiovascular Medicine, Mayo Clinic, Rochester, Minnesota

## Abstract

**Importance:**

Health-adjusted life expectancy, a measure of healthy longevity, lags longevity gains, resulting in a healthspan-lifespan gap.

**Objective:**

To quantify the healthspan-lifespan gap across the globe, investigate for sex disparities, and analyze morbidity and mortality associations.

**Design, Setting, and Participants:**

This retrospective cross-sectional study used the World Health Organization (WHO) Global Health Observatory as the global data source and acquired national-level data covering all continents. The 183 WHO member states were investigated. Statistical analysis was conducted from January to May 2024.

**Exposures:**

Data represent 2 decades of longitudinal follow-up.

**Main Outcomes and Measures:**

Changes in life expectancy and health-adjusted life expectancy, as well as the healthspan-lifespan gap were quantified for all participating member states. Gap assessment was stratified by sex. Correlations of the gap with morbidity and mortality were examined.

**Results:**

The healthspan-lifespan gap has widened globally over the last 2 decades among 183 WHO member states, extending to 9.6 years. A sex difference was observed with women presenting a mean (SD) healthspan-lifespan gap of 2.4 (0.5) years wider than men (*P* < .001). Healthspan-lifespan gaps were positively associated with the burden of noncommunicable diseases and total morbidity, and negatively with mortality. The US presented the largest healthspan-lifespan gap, amounting to 12.4 years, underpinned by a rise in noncommunicable diseases.

**Conclusions and Relevance:**

This study identifies growing healthspan-lifespan gaps around the globe, threatening healthy longevity across worldwide populations. Women globally exhibited a larger healthspan-lifespan gap than men.

## Key Points

**Question:**

What is the healthspan-lifespan gap, representing the number of years burdened by disease, in men and women across the world?

**Findings:**

This cross-sectional study quantified healthspan-lifespan gaps among 183 World Health Organization member states. Globally, the mean healthspan-lifespan gap was 9.6 years, and women exhibited a mean 2.4-year larger gap than men, associated with a disproportionately larger burden of noncommunicable diseases in women.

**Meaning:**

These findings suggest that the healthspan-lifespan gap is a universal threat to healthy longevity.

## Teaser

This cross-sectional study quantifies the healthspan-lifespan gap around the world, assesses sex disparities, and analyzes associations with morbidity and mortality.

## Introduction

Gains in life expectancy across global populations are recognized as a societal achievement.^1^ Increased lifespan, however, does not necessarily mean a longer healthy life.^2^ In considering quality of life, healthy longevity is increasingly underscored.^3^ To this end, characterizing healthspan—years lived in good health—would be valuable.^1,2^

An estimate of healthspan is the health-adjusted life expectancy whereby years of life are weighted by health status.^4^ Health-adjusted life expectancy estimates healthy longevity offering, a summary indicator of health and quality of life.^5,6^ Notably, gains in life expectancy have not been matched by an equivalent rise in health-adjusted life expectancy.^1^ The resulting healthspan-lifespan gap reflects the extent of lifespan burdened by disease.^1,2^ This longevity challenge warrants further study.^7^

We report here the healthspan-lifespan gaps for 183 World Health Organization (WHO) member states and investigate associations with longevity and disease burden. For each surveilled country, we also assess for presence of sex-disparity in healthspan-lifespan gaps.

## Methods

### Data Source

The cross-sectional study was exempt from institutional review board review and informed consent was not required as aggregate publicly available deidentified datasets were used, in accordance with 45 CFR §46. This study followed the Strengthening the Reporting of Observational Studies in Epidemiology (STROBE) reporting guideline for cross-sectional studies. The WHO Global Health Observatory provides a standardized and accessible resource of global health-related statistics,^8^ and was here queried in the first quarter of 2024.^9,10^ This study assessed changes in life expectancy, health-adjusted life expectancy, years lived with disability, and years of life lost among member states between the years of 2000 to 2019. Health-adjusted life expectancy was defined using Sullivan method^11^ as follows:



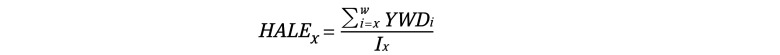



where *HALE_x_* represents health-adjusted life expectancy at age *x*, *YWD_i_* represents the years without disability between ages *x* and *x* + 5, and *I_x_* represents the number of people alive at age *x*. The WHO nomenclature was here adopted to designate individual countries or territories. The WHO burden of disease categories used *International Statistical Classification of Diseases and Related Health Problems, Tenth Revision*. Per capita disease burden was computed per 100 000 persons.

### Statistical Analysis

Statistical analysis was performed from January to May 2024 using the programming language R version 4.3.1 (R Project for Statistical Computing). The healthspan-lifespan gap was calculated by subtracting health-adjusted life expectancy from life expectancy.^1^

Kernel density estimates of the distribution of health-adjusted life expectancy and life expectancy were computed with the R package ggplot2. The mean rate of change in life expectancy and health-adjusted life expectancy were calculated as (*Estimate_2019_* − *Estimate_2000_*)/20, where *Estimate_2019_* represents either life expectancy or health-adjusted life expectancy in the year 2019 and *Estimate_2000_* represents either life expectancy or health-adjusted life expectancy in the year 2000. The association between life expectancy and the healthspan-lifespan gap was visualized by a Bland-Altman plot^12^ where the x-axis represents the mean of healthspan and lifespan for a member state and the y-axis represents the healthspan-lifespan gap. The 95% CI was calculated as *x* ± 1.96 × *SD_x_*, where *x* represents the mean healthspan-lifespan gap across all WHO surveilled member states and *SD_x_* represents the SD of the healthspan lifespan gap. The correlations between the healthspan-lifespan gap with years lived with disability attributable to noncommunicable diseases, total years lived with disability, and total years of life lost were estimated with linear regression with slopes (β), *P* values, and correlation coefficients (*R*^2^) reported. Likewise, correlations between sex dependent differences in the healthspan-lifespan gap (ie, women gap–men gap), noncommunicable disease burden and life expectancy were investigated via linear regression. Inferential statistics were conducted with Welch *t* test, Student *t* test, or Mann-Whitney *U* test. Values for mean (SD) are reported. Statistical significance was defined as 2-tailed *P* < .05.

## Results

### Life Expectancy and Health-Adjusted Life Expectancy Trends

**Figure 1.**
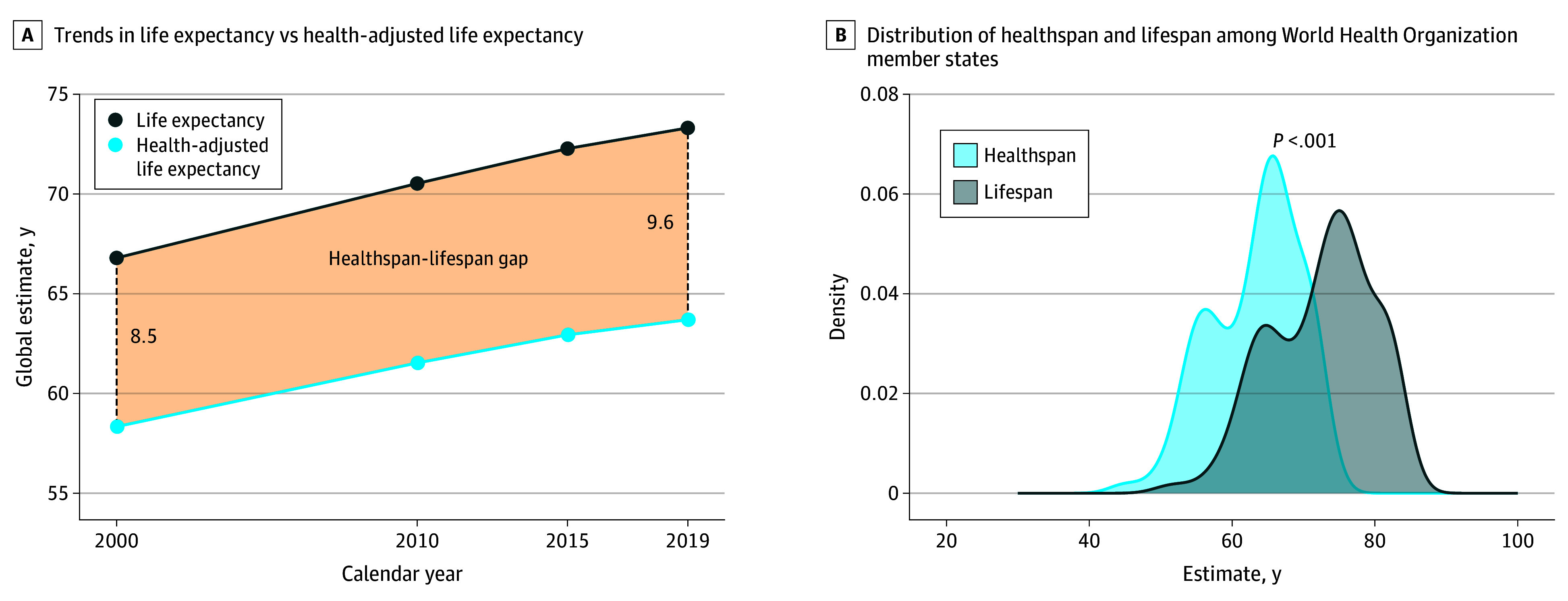
Global Life Expectancy, Health-Adjusted Life Expectancy, and Healthspan-Lifespan Gap A, Trends of life expectancy, health-adjusted life expectancy, and healthspan-lifespan gap. B, Distribution of healthspan and lifespan among 183 World Health Organization member states.

Over the last 2 decades, global life expectancy increased 6.5 years compared with the 5.4-year increase in health-adjusted life expectancy (Figure 1A). Among the 183 WHO member states, the mean (SD) rate of lifespan increase (0.29 [0.20] years/calendar year) was not matched by an equivalent increase in healthspan (0.24 [0.18] years/calendar year) (*P* < .001) (eTable 1 in Supplement 1). The greatest increases in lifespan were observed in Rwanda, Malawi, Burundi, Ethiopia, and Zambia with a mean increase over 20 years of 1.1, 1.0, 1.0, 0.9, and 0.9 years/calendar year, respectively (eTable 1 in Supplement 1). In contrast, declines in lifespan were observed in the Dominican Republic and Venezuela with a mean change of −0.02 and −0.01 years/calendar year, respectively (eTable 1 in Supplement 1). The greatest increases in healthspan were documented in Rwanda, Malawi, Burundi, Uganda, and Ethiopia with mean increases of 1.0, 0.9, 0.9, 0.8, and 0.8 years/calendar year, respectively (eTable 1 in Supplement 1). The Dominican Republic and Venezuela showed healthspan regression with a mean change of −0.01 and −0.01 years/calendar year, respectively (eTable 1 in Supplement 1). Worldwide, life expectancy outpaced health-adjusted life expectancy.

### The Global Healthspan-Lifespan Gaps

**Figure 2.**
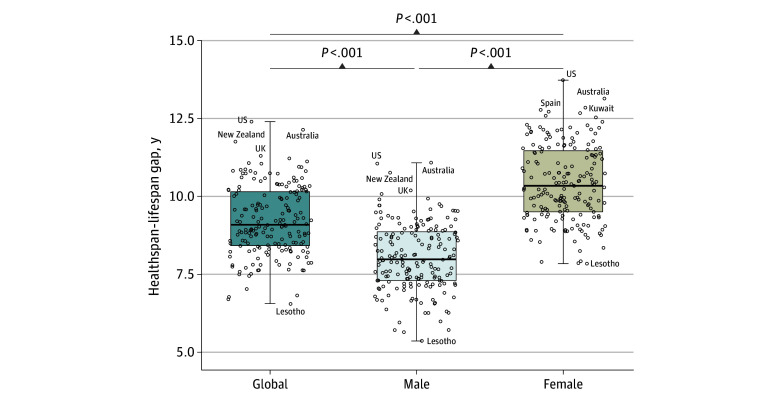
Healthspan-Lifespan Gaps for Individual World Health Organization Member States Stratified by Sex Boxplot represents 75th percentile, 50th percentile, and 25th percentile. Whiskers extend to the most extreme value within 1.5 times the IQR. Dots indicate individual countries.

The difference in life expectancy and health-adjusted life expectancy (ie, healthspan-lifespan gap) represents the number of years lived with disease or disability. Globally, an unequal rise in life expectancy compared to health-adjusted life expectancy resulted in a growing healthspan-lifespan gap climbing from 8.5 years in the year 2000 to 9.6 years in the year 2019, a 13% increase over the past 2 decades (Figure 1A). Across 183 WHO member states, the mean health-adjusted life expectancy of 63.3 years contrasted with a 72.5-year mean life expectancy (*P* < .001) (Figure 1B), underpinning a pangeographic lag in healthspan (eTable 2 in Supplement 1). The mean healthspan-lifespan gap across WHO member states was 9.2 years (Figure 2). The largest healthspan-lifespan gaps were observed in the US (12.4 years), Australia (12.1 years), New Zealand (11.8 years), United Kingdom of Great Britain and Northern Ireland (11.3 years), and Norway (11.2 years) (eTable 2 in Supplement 1). The smallest healthspan-lifespan gaps were observed in Lesotho (6.5 years), Central African Republic (6.7 years), Somalia (6.8 years), Kirbati (6.8 years), and Micronesia (7.0 years) (eTable 2 in Supplement 1). The healthspan-lifespan gap for each WHO member state is reported in eTable 2 in Supplement 1.

Globally, a mean (SD) difference of 2.4 (0.5) years between women and men in the healthspan-lifespan gap was observed (*P* < .001) (Figure 2). The largest sex disparities in the gap were in Germany (3.6 years), Spain (3.4 years), France (3.3 years), Portugal (3.2 years), and Lebanon (3.2 years) (eTable 2 in Supplement 1). The smallest sex inequalities were observed in Brunei Darussalam, Timor-Leste, Albania, Afghanistan, and Burundi with differences of 1.0, 1.3, 1.4, 1.4, and 1.4 years, respectively (eTable 2 in Supplement 1).

### Longevity and Noncommunicable Disease Burden Associated With Healthspan-Lifespan Gap

**Figure 3.**
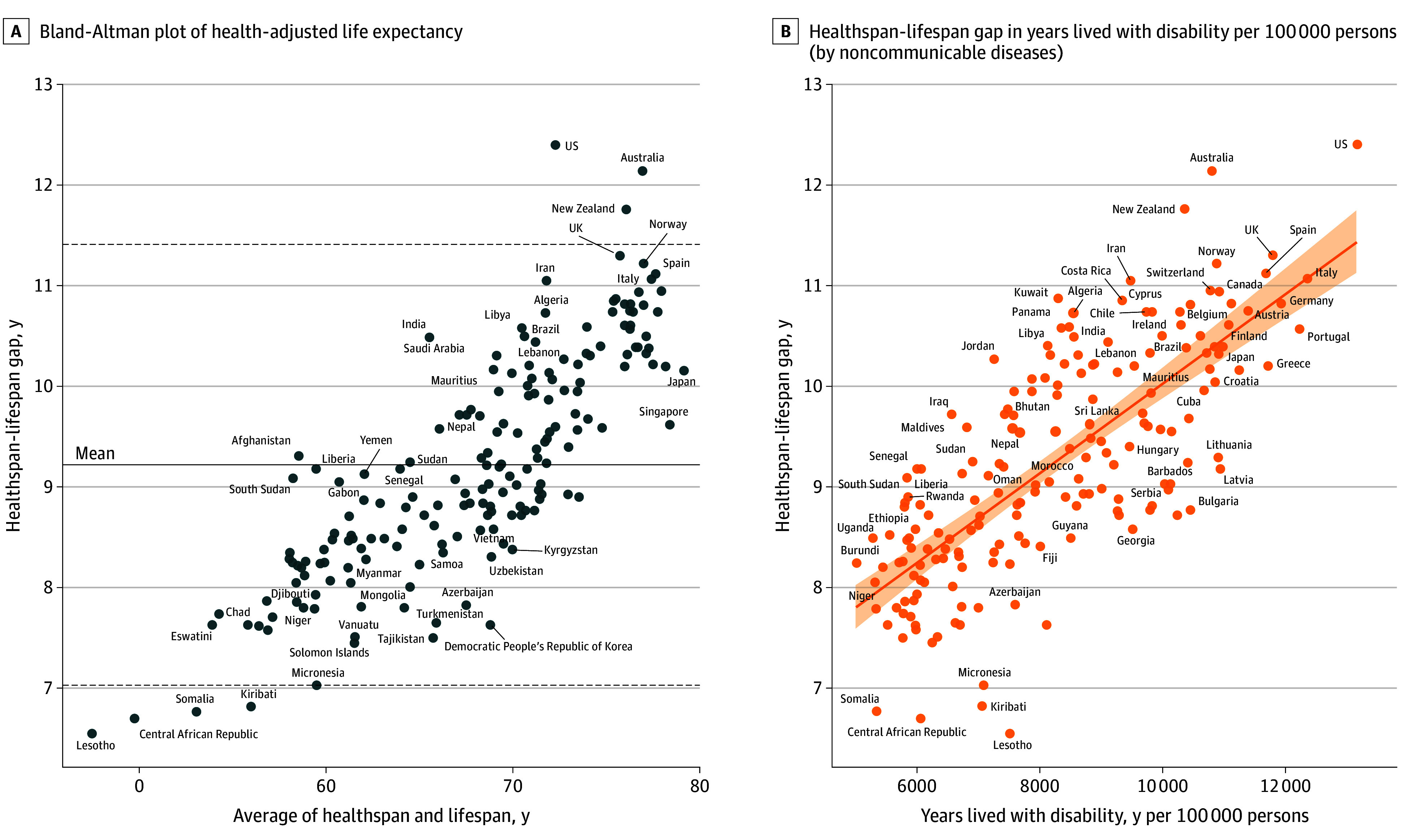
Healthspan-Lifespan Gap Associations With Life Expectancy and Disease Burden A, Bland-Altman plot of the health-adjusted life expectancy and life expectancy. Solid line represents the mean healthspan-lifespan gap. Dotted lines represent the 95% CI for the healthspan-lifespan gap. B, The healthspan-lifespan gap as a function of years lived with disability per 100 000 persons contributed by noncommunicable diseases. Solid line represents the line of best fit and the shaded ribbon represents the 95% CI for the regression line.

The healthspan-lifespan gap was positively associated with longevity as detected from the Bland-Altman plot (Figure 3A). The US, Australia, and New Zealand exhibited healthspan-lifespan gaps exceeding the upper limit of 95% CI (Figure 3A). In contrast, Lesotho, Central African Republic, Somalia, and Kiribati exhibited healthspan lifespan gaps below the lower limit of the 95% CI (Figure 3A).

The healthspan-lifespan gap was positively associated with morbidity burden assessed as total years lived with disability per 100 000 persons (β = 4.4 × 10^−4^; *R*^2^ = 0.42; *P* < .001) (eFigure 1 in Supplement 1) and was negatively associated with mortality burden estimated as total years of life lost per 100 000 persons (β = −6.6 × 10^−5^; *R*^2^ = 0.56; *P* < .001) (eFigure 2 in Supplement 1). In fact, the healthspan-lifespan gap correlated with the noncommunicable disease burden assessed as years lived with disability per 100 000 persons (β = 4.4 × 10^−4^; *R*^2^ = 0.55; *P* < .001) (Figure 3B). Sex disparity in the healthspan-lifespan gap was positively associated with sex disparity in the noncommunicable disease burden (β = 3.2 × 10^−4^; *R*^2^ = 0.22; *P* < .001) (eFigure 3 in Supplement 1) and a sex-dependent life expectancy difference (β = 0.11; *R*^2^ = 0.21; *P* < .001) with a mean (SD) longevity for women of 75.0 (7.1) years vs 70.1 (7.2) years for men (*P* < .001).

### US Trends

**Figure 4.**
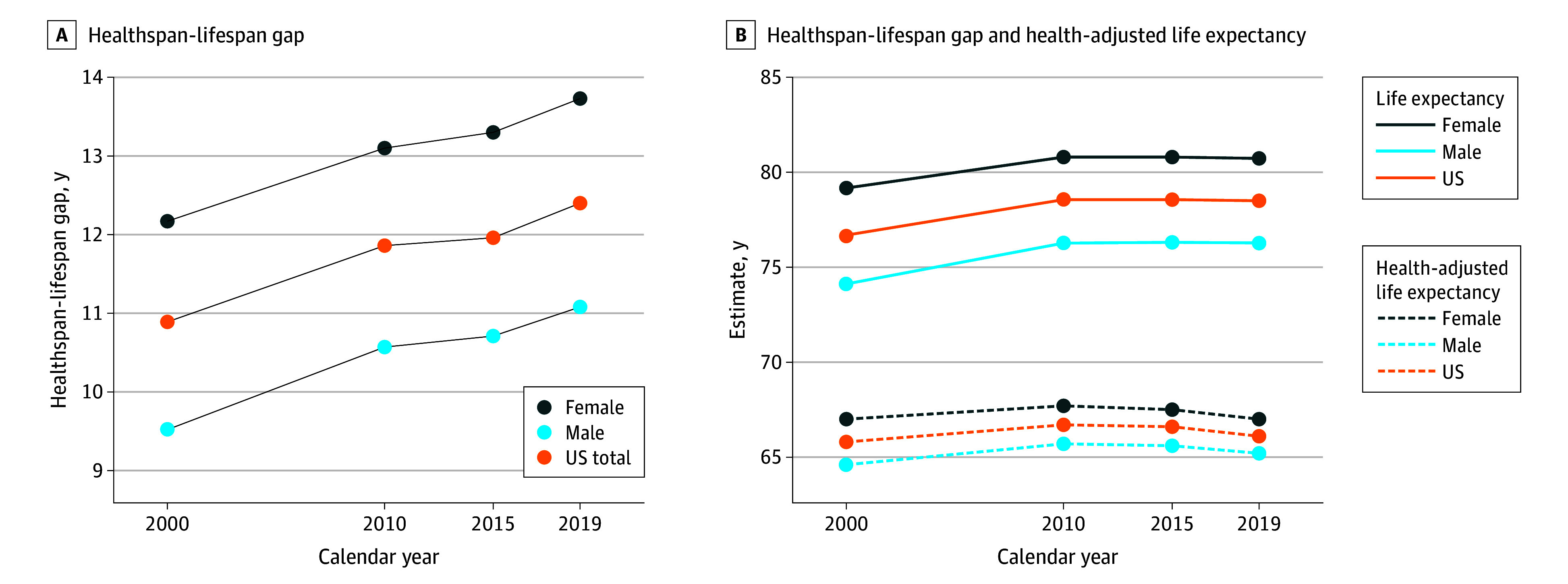
US Trends A, The US healthspan-lifespan gap with stratification by sex. B, US life expectancy and health-adjusted life expectancy with stratification by sex.

**Figure 5.**
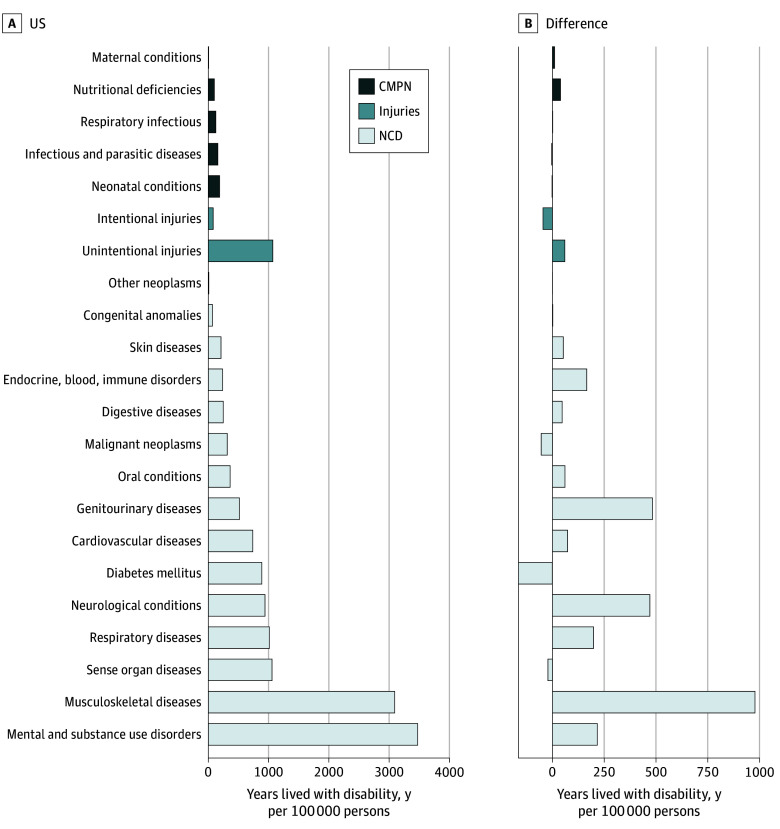
Years Lived With Disability Contributed by Disease Groups With Sex Differences in the US CMPN indicates communicable, maternal, perinatal and nutritional conditions; NCD, noncommunicable diseases.

Against the backdrop of the greatest noncommunicable disease burden, the US recorded the largest healthspan-lifespan gap (Figure 3B), with a gap 24% larger than projected from the country’s life expectancy. Specifically, in the US the mean healthspan-lifespan gap increased from 10.9 to 12.4 years over the past 2 decades (Figure 4A), resulting in a 29% higher gap than the global mean. Women exhibited a 2.6-year higher healthspan-lifespan gap than men, increasing from 12.2 to 13.7 years or 32% beyond the global mean for women (Figure 4A). In line with global trends, the gap in the US coincided with a disproportionate growth in life expectancy vs health-adjusted life expectancy. Life expectancy increased from 79.2 to 80.7 years in women, and from 74.1 to 76.3 years in men (Figure 4B). In contrast, health-adjusted life expectancy remained unchanged in women and increased 0.6 years in men (Figure 4B). The sex disparity in the gap (eTable 2 in Supplement 1) paralleled sex differences in noncommunicable disease burden with smaller contributions from communicable, maternal, perinatal, and nutritional conditions and injuries (eFigure 4 in Supplement 1). Mental and substance use disorders, along with musculoskeletal diseases, contributed most to years lived with disability in the US, whereas musculoskeletal, genitourinary, and neurological diseases contributed most to the differential burden in women (Figure 5).

## Discussion

This study reported life expectancy and health-adjusted life expectancy trends over the past 2 decades and found healthspan-lifespan gaps for each of the 183 WHO member states. Sex disparities in healthspan-lifespan gaps and association with longevity and disease burden are also reported. The US stands out with the largest healthspan-lifespan gap and the greatest noncommunicable disease burden. The risk to healthspan is found amplified by longevity and is here recognized to be more pronounced in women. The widening healthspan-lifespan gap is a global trend, as documented herein, and points to the need for an accelerated pivot to proactive wellness-centric care systems.^13^

The reporting of health-adjusted life expectancy has enabled tracking of healthy longevity^14^ in a systematic and uniform way, particularly relevant in the current era of a demographic longevity transition.^15^ In this context, the United Nations Decade of Healthy Aging aspires to evolve societal focus from longevity to healthy longevity.^15,16,17^ Accordingly, health-adjusted life expectancy is increasingly adopted as a valuable metric for tracking global health.^18^ Of note, health-adjusted life expectancy assesses the number of years people live free from disease, without considering the number of years people are burdened by disease. The present study refines the global health readout by introducing the projected years lived with disease, informing of a growing global threat to healthy longevity.

The trends registered across the 2 decades of surveillance, and reported here, highlight a chasm between advances made in longevity, a traditional measure of life expectancy, and healthy longevity, a contemporary indicator of quantity and quality of life.^19^ The widening healthspan gap reflects a growing number of years lived with disease that undermines success achieved with life expectancy, mandating measures that would narrow the widening gap.^1,20^ Strategies to address the healthspan-lifespan gap are multifaceted, leveraging preventative and curative solutions.^1,15,20,21^

The gap was found to be associated with morbidity burden and inversely associated with mortality. The growing incongruity between longevity and healthy longevity implicates a disease paradox whereby reduced acute mortality exposes survivors to an increased burden of chronic disease.^22,23,24^ As shown here, mortality rates negatively associated with healthspan-lifespan gaps. Mental and substance use disorders, along with musculoskeletal diseases, contributed most to the disease burden in the US. Identifying contributors to the gap unique to each geography can help inform prioritization of interventional domains specific to countries and regions.^25^

A sex disparity in the healthspan-lifespan gap was identified here at global scale, partly underpinned by the higher life expectancy in women^26^ and a distinctly higher noncommunicable disease burden. The US healthspan-lifespan sex disparity correlated with a much higher per capita women musculoskeletal burden in line with a higher musculoskeletal disease burden globally in women.^27^ Indeed, while the healthspan-lifespan gap is a universal challenge requiring transnational consideration, country-specific profiles invite tailored interventions to maximize equitable and sustainable healthy aging.

### Limitations

This study had limitations. The healthspan-lifespan gap reflects the number of years lived with disease, dependent on estimates of life expectancy and health-adjusted life expectancy.^4^ Health-adjusted life expectancy calculations estimate the mean number of years lived in full health and thus rely on disability weights assigned to various health conditions.^28^ These weights have been revised through surveys of diverse populations to reflect multicultural perceptions,^29^ yet may be impacted by survey methods or overrepresentation of unaffected individuals.^30^

## Conclusions

Surveillance of healthspan-lifespan gaps among 183 WHO member states found a widening healthspan-lifespan gap worldwide. A larger healthspan-lifespan gap was observed in women. The US stood out with the largest healthspan-lifespan gap and greatest noncommunicable disease burden. These results underscore that around the world, while people live longer, they live a greater number of years burdened by disease. To identify drivers of the healthspan-lifespan gap, associated demographic, health, and economic characteristics need to be investigated by geography.

## Notes

Supplement 1. **eTable 1.** Average Rate of Change in Life Expectancy and Health-Adjusted Life Expectancy Among World Health Organization Member States Between 2000 and 2019 **eTable 2.** The 2019 Healthspan-Lifespan Gap Across World Health Organization Member States Stratified by Sex **eFigure 1.** Healthspan-Lifespan Gap for 183 World Health Organization Member States as a Function of Total Years Lived With Disability per 100,000 Persons **eFigure 2.** Healthspan-Lifespan Gaps for 183 World Health Organization Member States as a Function of Total Years of Life Lost per 100,000 Persons **eFigure 3.** The Sex Disparity (Women-Men) in the Healthspan-Lifespan Gap in 2019 as a Function of The Sex Disparity (Women-Men) in Years Lived With Disability (YLD) for Noncommunicable Diseases (NCD) per 100,000 Persons **eFigure 4.** Years Lived With Disability (YLD) for Noncommunicable Diseases (NCD) and Injuries, and Communicable, Maternal, Perinatal and Nutritional Conditions (CMPN) for the United States of America Total Population, Men, and Women

Supplement 2.
Data Sharing Statement
