# Supplementary material for: Global Healthspan-Lifespan Gaps Among 183 World Health Organization Member States
Source: JAMA Netw Open. 2024 Dec 11;7(12):e2450241. doi: 10.1001/jamanetworkopen.2024.50241 (PMC11635540; doi:10.1001/jamanetworkopen.2024.50241)
Supplement: Supplement 1. — eTable 1. Average Rate of Change in Life Expectancy and Health-Adjusted Life Expectancy Among World Health Organization Member States Between 2000 and 2019 eTable 2. The 2019 Healthspan-Lifespan Gap Across World Health Organization Member States Stratified by Sex eFigure 1. Healthspan-Lifespan Gap for 183 World Health Organization Member States as a Function of Total Years Lived With Disability per 100,000 Persons eFigure 2. Healthspan-Lifespan Gaps for 183 World Health Organization Member States as a Function of Total Years of Life Lost per 100,000 Persons eFigure 3. The Sex Disparity (Women-Men) in the Healthspan-Lifespan Gap in 2019 as a Function of The Sex Disparity (Women-Men) in Years Lived With Disability (YLD) for Noncommunicable Diseases (NCD) per 100,000 Persons eFigure 4. Years Lived With Disability (YLD) for Noncommunicable Diseases (NCD) and Injuries, and Communicable, Maternal, Perinatal and Nutritional Conditions (CMPN) for the United States of America Total Population, Men, and Women [file jamanetwopen-e2450241-s001.pdf]

Supplemental Online Content

Garmany A, Terzic A. Global healthspan-lifespan gaps among 183 World Health Organization member states. *JAMA Netw Open.* 2024;7(12):e2450241. doi:10.1001/jamanetworkopen.2024.50241

eTable 1. Average Rate of Change in Life Expectancy and Health-Adjusted Life Expectancy Among World Health Organization Member States Between 2000 and 2019

eTable 2. The 2019 Healthspan-Lifespan Gap Across World Health Organization Member States Stratified by Sex

eFigure 1. Healthspan-Lifespan Gap for 183 World Health Organization Member States as a Function of Total Years Lived With Disability per 100,000 Persons

eFigure 2. Healthspan-Lifespan Gaps for 183 World Health Organization Member States as a Function of Total Years of Life Lost per 100,000 Persons

eFigure 3. The Sex Disparity (Women-Men) in the Healthspan-Lifespan Gap in 2019 as a Function of The Sex Disparity (Women-Men) in Years Lived With Disability (YLD) for Noncommunicable Diseases (NCD) per 100,000 Persons

eFigure 4. Years Lived With Disability (YLD) for Noncommunicable Diseases (NCD) and Injuries, and Communicable, Maternal, Perinatal and Nutritional Conditions (CMPN) for the United States of America Total Population, Men, and Women

This supplemental material has been provided by the authors to give readers additional information about their work.

© 2024 Garmany A et al. *JAMA Network Open.*

eTable 1. Average Rate of Change in Life Expectancy and Health-Adjusted Life Expectancy Among World Health Organization Member States Between 2000 and 2019.

World Health Organization Member State	Average Change in Life Expectancy (years/calendar year)	Average Change in Health-Adjusted Life Expectancy (years/calendar year)
Afghanistan	0.41	0.36
Albania	0.22	0.19
Algeria	0.25	0.18
Angola	0.69	0.60
Antigua and Barbuda	0.09	0.07
Argentina	0.12	0.10
Armenia	0.21	0.18
Australia	0.17	0.12
Austria	0.17	0.14
Azerbaijan	0.30	0.26
Bahamas	0.11	0.08
Bahrain	0.27	0.20
Bangladesh	0.43	0.36
Barbados	0.08	0.05
Belarus	0.30	0.26
Belgium	0.19	0.14
Belize	0.20	0.15
Benin	0.34	0.30
Bhutan	0.37	0.31
Bolivia	0.31	0.27
Bosnia and Herzegovina	0.07	0.05

© 2024 Garmany A et al. *JAMA Network Open*.

Botswana	0.83	0.69
Brazil	0.22	0.19
Brunei Darussalam	0.11	0.09
Bulgaria	0.17	0.14
Burkina Faso	0.55	0.50
Burundi	1.00	0.87
Cabo Verde	0.15	0.14
Cambodia	0.57	0.51
Cameroon	0.48	0.42
Canada	0.16	0.09
Central African Republic	0.44	0.38
Chad	0.43	0.37
Chile	0.20	0.15
China	0.29	0.24
Colombia	0.28	0.24
Comoros	0.29	0.26
Congo	0.63	0.54
Costa Rica	0.14	0.10
Cote d'Ivoire	0.63	0.54
Croatia	0.21	0.16
Cuba	0.05	0.04
Cyprus	0.22	0.16
Czechia	0.21	0.14
Democratic People's Republic of Korea	0.43	0.36
Democratic Republic of the Congo	0.50	0.44
Denmark	0.22	0.17
Djibouti	0.30	0.26
Dominican Republic	-0.02	-0.01

© 2024 Garmany A et al. *JAMA Network Open*.

Ecuador	0.20	0.16
Egypt	0.10	0.09
El Salvador	0.13	0.11
Equatorial Guinea	0.39	0.35
Eritrea	0.48	0.44
Estonia	0.40	0.33
Eswatini	0.53	0.44
Ethiopia	0.91	0.78
Fiji	0.11	0.08
Finland	0.20	0.16
France	0.18	0.14
Gabon	0.42	0.35
Gambia	0.31	0.27
Georgia	0.19	0.14
Germany	0.18	0.12
Ghana	0.35	0.30
Greece	0.15	0.11
Grenada	0.05	0.02
Guatemala	0.26	0.22
Guinea	0.34	0.29
Guinea-Bissau	0.50	0.43
Guyana	0.14	0.11
Haiti	0.35	0.29
Honduras	0.09	0.09
Hungary	0.26	0.21
Iceland	0.13	0.11
India	0.43	0.37
Indonesia	0.21	0.19

© 2024 Garmany A et al. *JAMA Network Open*.

Iran	0.24	0.18
Iraq	0.18	0.16
Ireland	0.27	0.20
Israel	0.20	0.16
Italy	0.18	0.14
Jamaica	0.09	0.06
Japan	0.16	0.13
Jordan	0.26	0.20
Kazakhstan	0.54	0.44
Kenya	0.61	0.54
Kiribati	0.18	0.15
Kuwait	0.16	0.12
Kyrgyzstan	0.41	0.36
Lao People's Democratic Republic	0.51	0.44
Latvia	0.26	0.23
Lebanon	0.09	0.06
Lesotho	0.15	0.11
Liberia	0.52	0.45
Libya	0.07	0.03
Lithuania	0.20	0.18
Luxembourg	0.21	0.16
Madagascar	0.33	0.30
Malawi	1.04	0.90
Malaysia	0.10	0.08
Maldives	0.43	0.38
Mali	0.51	0.45
Malta	0.20	0.15
Mauritania	0.32	0.27

© 2024 Garmany A et al. *JAMA Network Open*.

Mauritius	0.14	0.10
Mexico	0.08	0.06
Micronesia	0.09	0.07
Mongolia	0.38	0.32
Montenegro	0.12	0.09
Morocco	0.18	0.15
Mozambique	0.37	0.34
Myanmar	0.46	0.40
Namibia	0.56	0.47
Nepal	0.28	0.25
Netherlands	0.19	0.13
New Zealand	0.17	0.12
Nicaragua	0.09	0.09
Niger	0.65	0.57
Nigeria	0.45	0.40
North Macedonia	0.19	0.15
Norway	0.20	0.15
Oman	0.24	0.19
Pakistan	0.28	0.23
Panama	0.10	0.07
Papua New Guinea	0.10	0.09
Paraguay	0.05	0.04
Peru	0.27	0.23
Philippines	0.07	0.07
Poland	0.23	0.18
Portugal	0.25	0.21
Qatar	0.29	0.23
Republic of Korea	0.35	0.28

© 2024 Garmany A et al. *JAMA Network Open*.

Republic of Moldova	0.32	0.26
Romania	0.21	0.18
Russian Federation	0.40	0.34
Rwanda	1.08	0.95
Saint Lucia	0.06	0.03
Saint Vincent and the Grenadines	0.13	0.09
Samoa	0.10	0.07
Sao Tome and Principe	0.35	0.30
Saudi Arabia	0.19	0.14
Senegal	0.50	0.43
Serbia	0.22	0.17
Seychelles	0.07	0.05
Sierra Leone	0.60	0.53
Singapore	0.24	0.20
Slovakia	0.25	0.19
Slovenia	0.26	0.21
Solomon Islands	0.14	0.10
Somalia	0.33	0.29
South Africa	0.48	0.38
South Sudan	0.43	0.36
Spain	0.21	0.15
Sri Lanka	0.25	0.20
Sudan	0.33	0.28
Suriname	0.08	0.05
Sweden	0.14	0.10
Switzerland	0.19	0.16
Syrian Arab Republic	0.06	0.00
Tajikistan	0.20	0.18

© 2024 Garmany A et al. *JAMA Network Open*.

Thailand	0.32	0.28
Timor-Leste	0.35	0.39
Togo	0.42	0.36
Tonga	0.08	0.06
Trinidad and Tobago	0.32	0.24
Tunisia	0.15	0.10
Turkiye	0.21	0.17
Turkmenistan	0.32	0.27
Uganda	0.90	0.78
Ukraine	0.27	0.23
United Arab Emirates	0.14	0.11
United Kingdom	0.18	0.13
United Republic of Tanzania	0.74	0.65
United States of America	0.09	0.02
Uruguay	0.11	0.08
Uzbekistan	0.39	0.33
Vanuatu	0.07	0.05
Venezuela	-0.01	-0.01
Viet Nam	0.11	0.10
Yemen	0.20	0.15
Zambia	0.90	0.77
Zimbabwe	0.71	0.60

© 2024 Garmany A et al. *JAMA Network Open*.

eTable 2. The 2019 Healthspan-Lifespan Gap Across World Health Organization Member States Stratified by Sex.

World Health Organization Member State	Healthspan-Lifespan Gap Both Sexes (years)	Healthspan-Lifespan Gap Men (years)	Healthspan-Lifespan Gap Women (years)	Sex Disparity in Healthspan-Lifespan Gap (years)
Afghanistan	9.31	8.59	9.96	1.37
Albania	8.90	8.25	9.61	1.36
Algeria	10.73	9.53	12.02	2.49
Angola	8.26	7.10	9.32	2.22
Antigua and Barbuda	9.45	8.68	10.26	1.58
Argentina	9.48	8.11	10.70	2.59
Armenia	8.93	7.59	10.06	2.47
Australia	12.14	11.05	13.14	2.09
Austria	10.75	9.54	11.88	2.34
Azerbaijan	7.83	6.68	8.89	2.21
Bahamas	8.81	7.55	10.09	2.54
Bahrain	9.91	9.04	11.52	2.48
Bangladesh	9.95	8.79	11.24	2.45
Barbados	9.03	8.12	9.96	1.84
Belarus	8.81	7.35	10.22	2.87
Belgium	10.82	9.49	12.21	2.72
Belize	9.11	7.86	10.47	2.61
Benin	7.93	6.69	9.06	2.37
Bhutan	9.71	8.83	10.89	2.06
Bolivia	8.84	7.95	9.83	1.88
Bosnia and Herzegovina	9.55	8.68	10.39	1.71
Botswana	8.35	7.05	9.66	2.61
Brazil	10.50	9.05	11.99	2.94

© 2024 Garmany A et al. *JAMA Network Open*.

Brunei Darussalam	8.72	8.19	9.26	1.07
Bulgaria	8.77	7.75	9.90	2.15
Burkina Faso	7.80	6.66	8.93	2.27
Burundi	8.24	7.55	8.94	1.39
Cabo Verde	9.23	7.70	10.74	3.04
Cambodia	8.62	7.43	9.75	2.32
Cameroon	7.86	6.79	8.90	2.11
Canada	10.94	9.90	12.05	2.15
Central African Republic	6.70	5.71	7.86	2.15
Chad	7.63	6.65	8.54	1.89
Chile	10.74	9.09	12.15	3.06
China	8.93	7.53	10.49	2.96
Colombia	10.31	9.29	11.37	2.08
Comoros	8.49	7.62	9.28	1.66
Congo	8.54	7.41	9.51	2.10
Costa Rica	10.85	9.71	12.14	2.43
Côte d'Ivoire	8.12	7.13	9.31	2.18
Croatia	10.04	8.84	11.10	2.26
Cuba	9.96	8.77	11.05	2.28
Cyprus	10.74	9.32	12.12	2.80
Czechia	10.33	9.30	11.33	2.03
Democratic People's Republic of Korea	7.63	5.99	9.09	3.10
Democratic Republic of the Congo	8.25	7.20	9.42	2.22
Denmark	10.32	8.89	11.62	2.73
Djibouti	7.81	6.90	8.88	1.98
Dominican Republic	8.84	7.66	10.09	2.43

© 2024 Garmany A et al. *JAMA Network Open*.

Ecuador	9.95	8.71	11.20	2.49
Egypt	8.82	7.29	10.44	3.15
El Salvador	10.13	8.98	11.33	2.35
Equatorial Guinea	8.29	7.47	9.48	2.01
Eritrea	8.38	7.40	9.37	1.97
Estonia	9.68	8.30	10.90	2.60
Eswatini	7.63	6.26	9.38	3.12
Ethiopia	8.80	7.90	9.72	1.82
Fiji	8.41	7.43	9.58	2.15
Finland	10.61	9.26	12.04	2.78
France	10.38	8.66	11.99	3.33
Gabon	8.87	7.59	10.43	2.84
Gambia	8.47	7.02	9.95	2.93
Georgia	8.58	7.39	9.86	2.47
Germany	10.82	9.02	12.67	3.65
Ghana	8.28	7.16	9.56	2.40
Greece	10.20	8.74	11.67	2.93
Grenada	8.98	8.03	9.93	1.90
Guatemala	9.72	8.45	10.94	2.49
Guinea	7.71	6.58	8.55	1.97
Guinea-Bissau	7.62	6.26	8.88	2.62
Guyana	8.49	7.35	9.69	2.34
Haiti	8.25	7.44	8.96	1.52
Honduras	8.94	7.97	9.86	1.89
Hungary	9.24	8.09	10.29	2.20
Iceland	10.33	9.11	11.57	2.46
India	10.49	9.22	11.77	2.55
Indonesia	8.51	7.50	9.50	2.00

© 2024 Garmany A et al. *JAMA Network Open*.

Iran	11.05	9.69	12.59	2.90
Iraq	9.72	8.33	11.27	2.94
Ireland	10.74	9.50	12.08	2.58
Israel	10.22	8.79	11.66	2.87
Italy	11.07	9.71	12.30	2.59
Jamaica	9.38	8.46	10.38	1.92
Japan	10.16	8.89	11.44	2.55
Jordan	10.27	8.92	11.58	2.66
Kazakhstan	8.95	7.58	10.21	2.63
Kenya	8.39	7.30	9.54	2.24
Kiribati	6.82	5.64	7.90	2.26
Kuwait	10.87	9.75	12.85	3.10
Kyrgyzstan	8.38	7.15	9.61	2.46
Lao People's Democratic Republic	8.01	6.99	9.05	2.06
Latvia	9.18	7.68	10.54	2.86
Lebanon	10.44	8.93	12.15	3.22
Lesotho	6.55	5.36	7.84	2.48
Liberia	9.18	8.25	10.00	1.75
Libya	10.58	9.31	11.84	2.53
Lithuania	9.29	7.83	10.73	2.90
Luxembourg	10.81	9.52	12.20	2.68
Madagascar	8.05	7.20	8.90	1.70
Malawi	8.52	7.21	9.93	2.72
Malaysia	9.02	8.11	10.18	2.07
Maldives	9.59	8.93	10.76	1.83
Mali	8.20	7.40	8.90	1.50
Malta	10.39	9.04	11.90	2.86

© 2024 Garmany A et al. *JAMA Network Open*.

Mauritania	8.58	7.88	9.33	1.45
Mauritius	10.17	8.95	11.42	2.47
Mexico	10.21	8.83	11.66	2.83
Micronesia	7.03	5.95	8.25	2.30
Mongolia	7.80	6.72	8.96	2.24
Montenegro	8.88	7.95	9.95	2.00
Morocco	9.29	7.98	10.61	2.63
Mozambique	7.74	6.56	8.93	2.37
Myanmar	8.23	7.11	9.40	2.29
Namibia	8.48	7.18	9.85	2.67
Nepal	9.58	8.28	10.65	2.37
Netherlands	10.39	9.10	11.65	2.55
New Zealand	11.76	10.76	12.72	1.96
Nicaragua	9.54	8.37	10.73	2.36
Niger	7.79	6.76	8.76	2.00
Nigeria	8.22	7.30	9.20	1.90
Norway	11.22	10.08	12.53	2.45
Oman	9.20	8.47	10.76	2.29
Pakistan	8.71	7.69	9.92	2.23
Panama	10.59	9.25	12.06	2.81
Papua New Guinea	8.20	7.20	9.26	2.06
Paraguay	10.01	8.58	11.55	2.97
Peru	10.40	9.26	11.54	2.28
Philippines	8.43	7.30	9.70	2.40
Poland	9.57	8.63	10.63	2.00
Portugal	10.57	8.96	12.20	3.24
Qatar	10.07	9.93	11.53	1.60
Republic of Korea	10.20	9.02	11.39	2.37

© 2024 Garmany A et al. *JAMA Network Open*.

Republic of Moldova	8.76	7.36	10.02	2.66
North Macedonia	8.72	7.74	9.57	1.83
Romania	8.77	7.65	9.86	2.21
Russian Federation	9.03	7.48	10.50	3.02
Rwanda	8.90	7.88	9.84	1.96
Saint Lucia	9.63	8.30	11.11	2.81
Saint Vincent and the Grenadines	9.22	8.42	10.22	1.80
Samoa	8.35	7.36	9.35	1.99
Sao Tome and Principe	8.82	7.89	9.79	1.90
Saudi Arabia	10.31	9.31	11.75	2.44
Senegal	9.18	8.02	10.24	2.22
Serbia	8.97	8.06	9.88	1.82
Seychelles	9.34	8.08	10.75	2.67
Sierra Leone	7.87	7.10	8.60	1.50
Singapore	9.62	8.65	10.75	2.10
Slovakia	9.73	8.64	10.64	2.00
Slovenia	10.61	9.59	11.56	1.97
Solomon Islands	7.45	6.37	8.77	2.40
Somalia	6.77	5.71	7.92	2.21
South Africa	9.05	7.60	10.59	2.99
South Sudan	9.09	7.93	10.34	2.41
Spain	11.12	9.38	12.78	3.40
Sri Lanka	9.87	8.70	10.81	2.11
Sudan	9.25	7.95	10.46	2.51
Suriname	9.08	7.80	10.43	2.63
Sweden	10.50	9.13	11.87	2.74
Switzerland	10.95	9.55	12.28	2.73

© 2024 Garmany A et al. *JAMA Network Open*.

Syrian Arab Republic	9.77	8.68	10.96	2.28
Tajikistan	7.50	6.68	8.35	1.67
Thailand	9.40	8.46	10.44	1.98
Timor-Leste	8.72	8.13	9.41	1.28
Togo	8.07	6.82	9.43	2.61
Tonga	8.57	7.11	10.11	3.00
Trinidad and Tobago	9.93	8.54	11.32	2.78
Tunisia	10.14	8.78	11.49	2.71
Türkiye	10.22	8.64	11.67	3.03
Turkmenistan	7.65	6.58	8.67	2.09
Uganda	8.49	7.24	9.70	2.46
Ukraine	8.72	7.40	10.01	2.61
United Arab Emirates	10.08	9.30	12.22	2.92
United Kingdom	11.30	10.19	12.39	2.20
United Republic of Tanzania	8.84	7.77	9.96	2.19
United States of America	12.40	11.08	13.73	2.65
Uruguay	9.60	8.08	11.06	2.98
Uzbekistan	8.31	7.26	9.41	2.15
Vanuatu	7.51	6.26	8.92	2.66
Venezuela	9.55	8.01	11.07	3.06
Viet Nam	8.44	7.16	9.81	2.65
Yemen	9.13	7.51	10.72	3.21
Zambia	8.05	7.04	9.07	2.03
Zimbabwe	7.58	6.31	8.81	2.50

© 2024 Garmany A et al. *JAMA Network Open*.

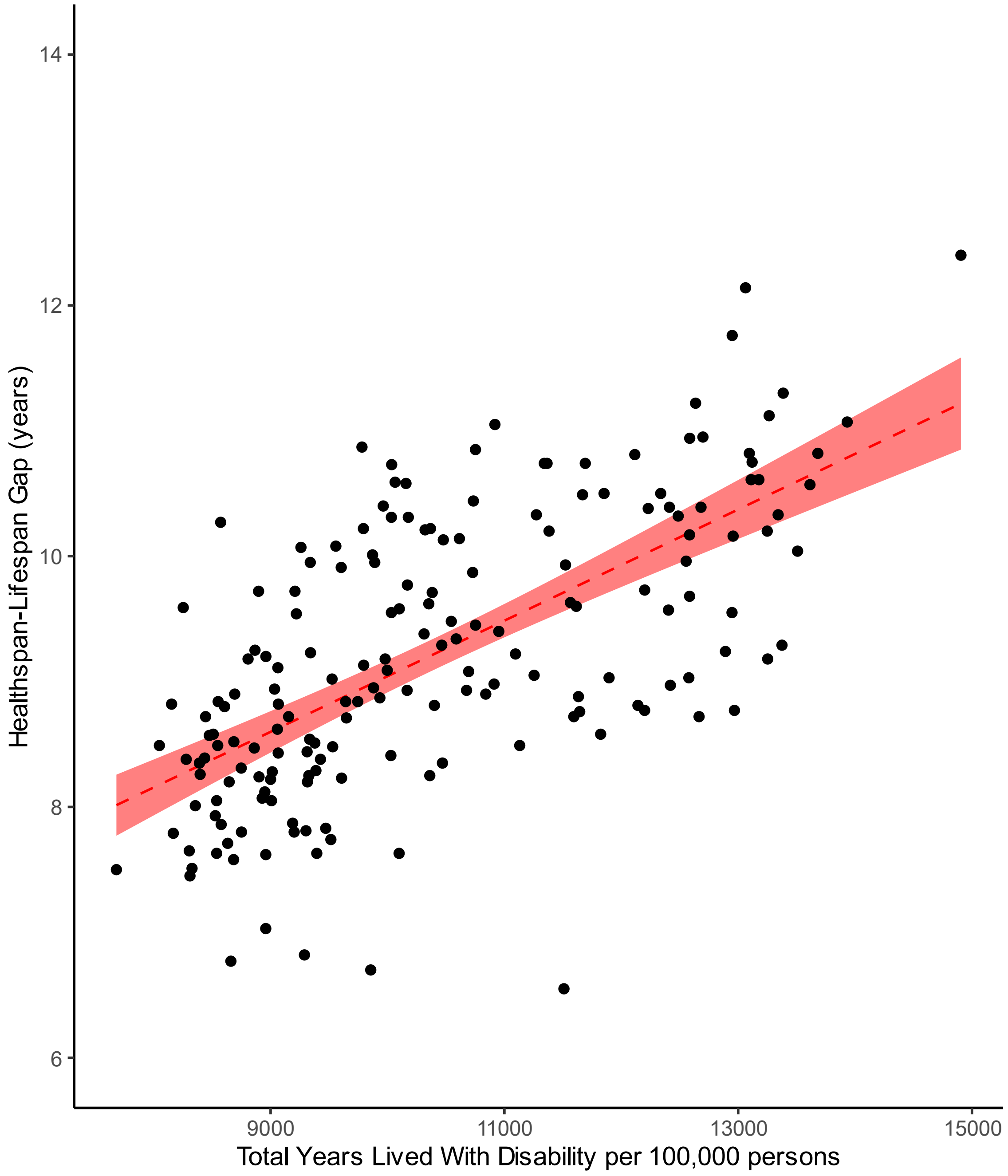

eFigure 1. Healthspan-lifespan gap for 183 World Health Organization member states as a function of total years lived with disability per 100,000 persons. Dashed line represents the line of best fit with the ribbon representing the 95% confidence interval.

© 2024 Garmany A et al. *JAMA Network Open*.

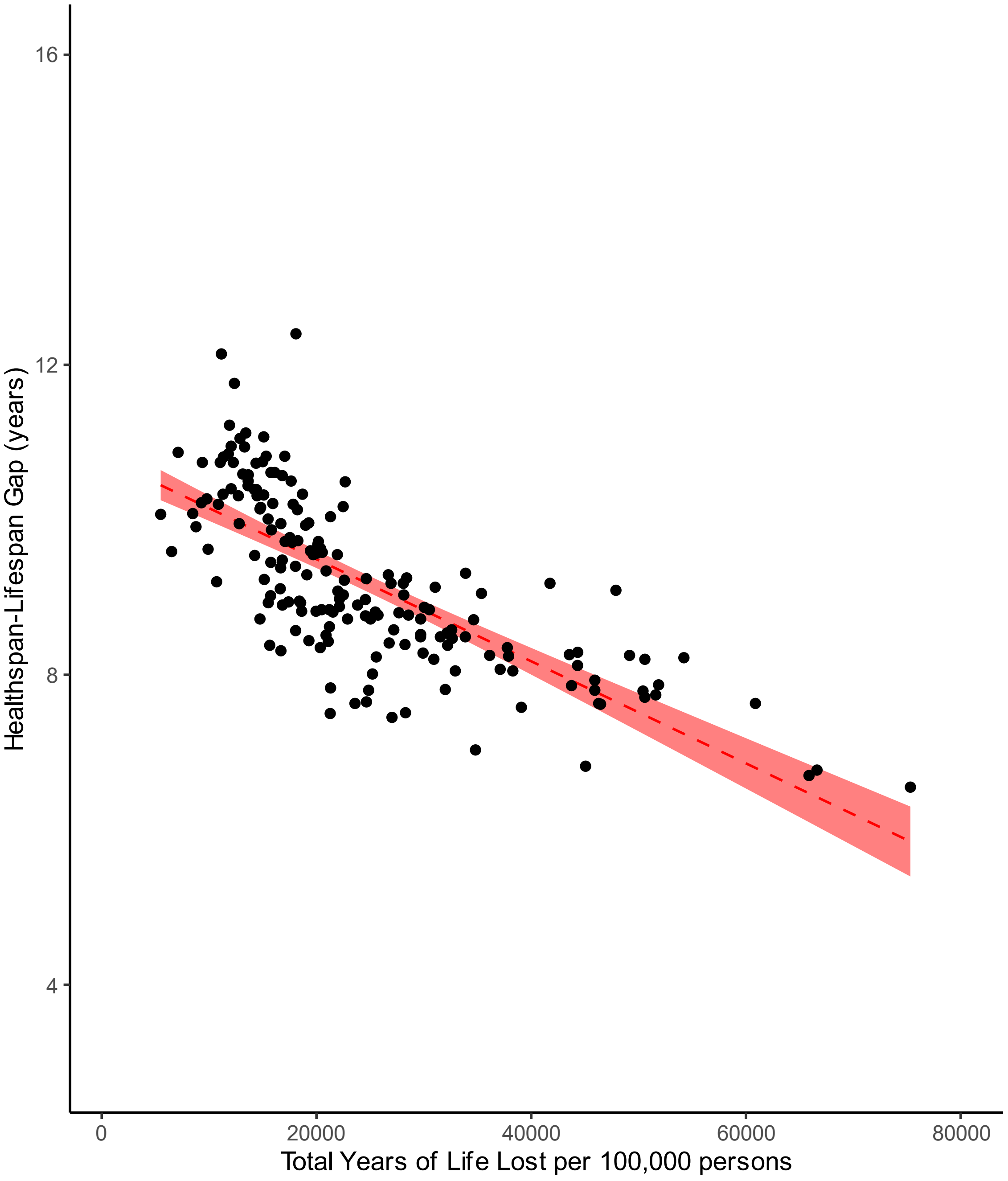

eFigure 2. Healthspan-lifespan gaps for 183 World Health Organization member states as a function of total years of life lost per 100,000 persons. Dashed line represents the line of best fit with the ribbon representing the 95% confidence interval.

© 2024 Garmany A et al. *JAMA Network Open*.

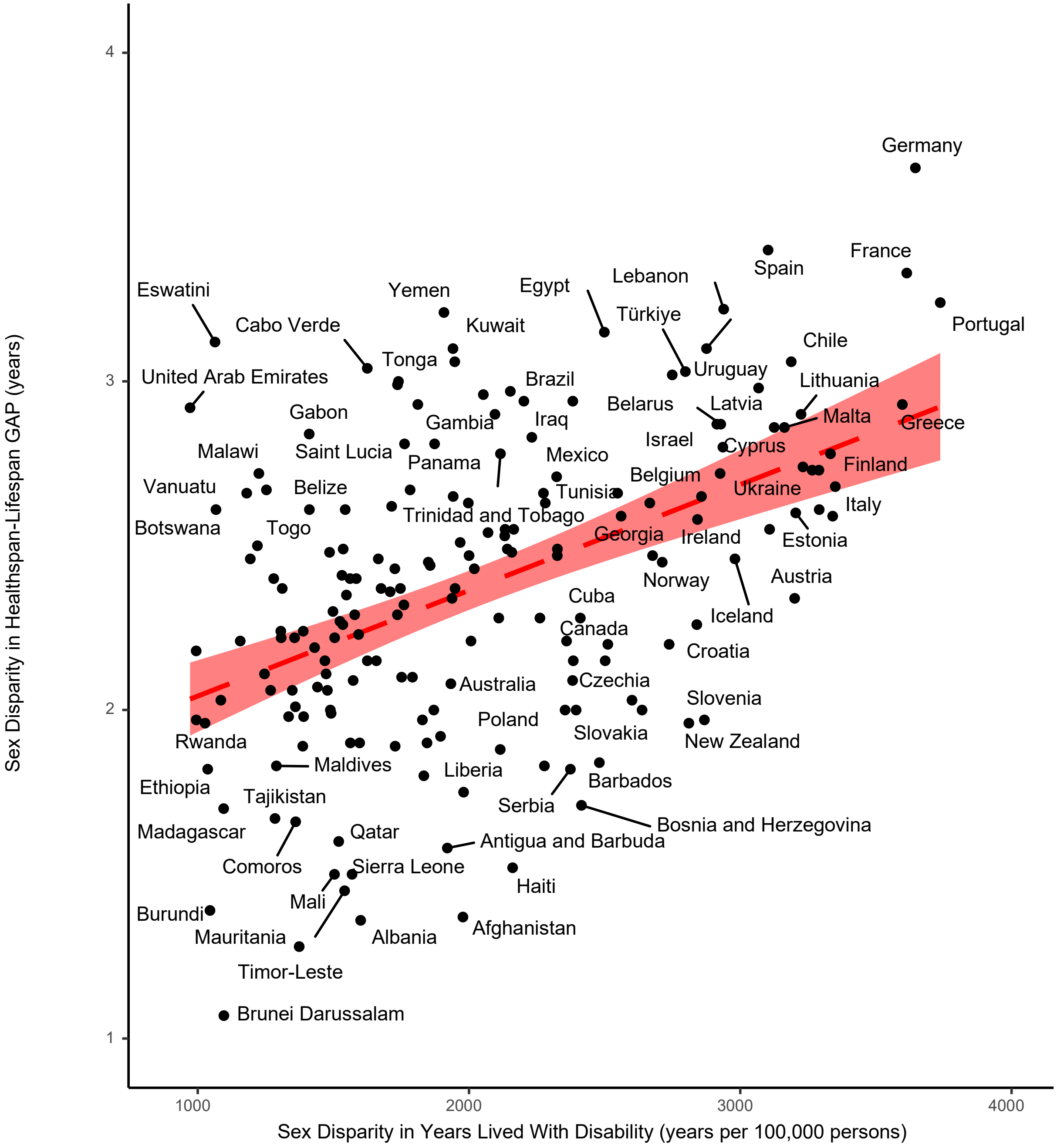

eFigure 3. The sex disparity (Women-Men) in the healthspan-lifespan gap in 2019 as a function of the sex disparity (Women-Men) in years lived with disability (YLD) for noncommunicable diseases (NCD) per 100,000 persons. Dashed line represents the line of best fit with the ribbon representing the 95% confidence interval.

© 2024 Garmany A et al. *JAMA Network Open*.

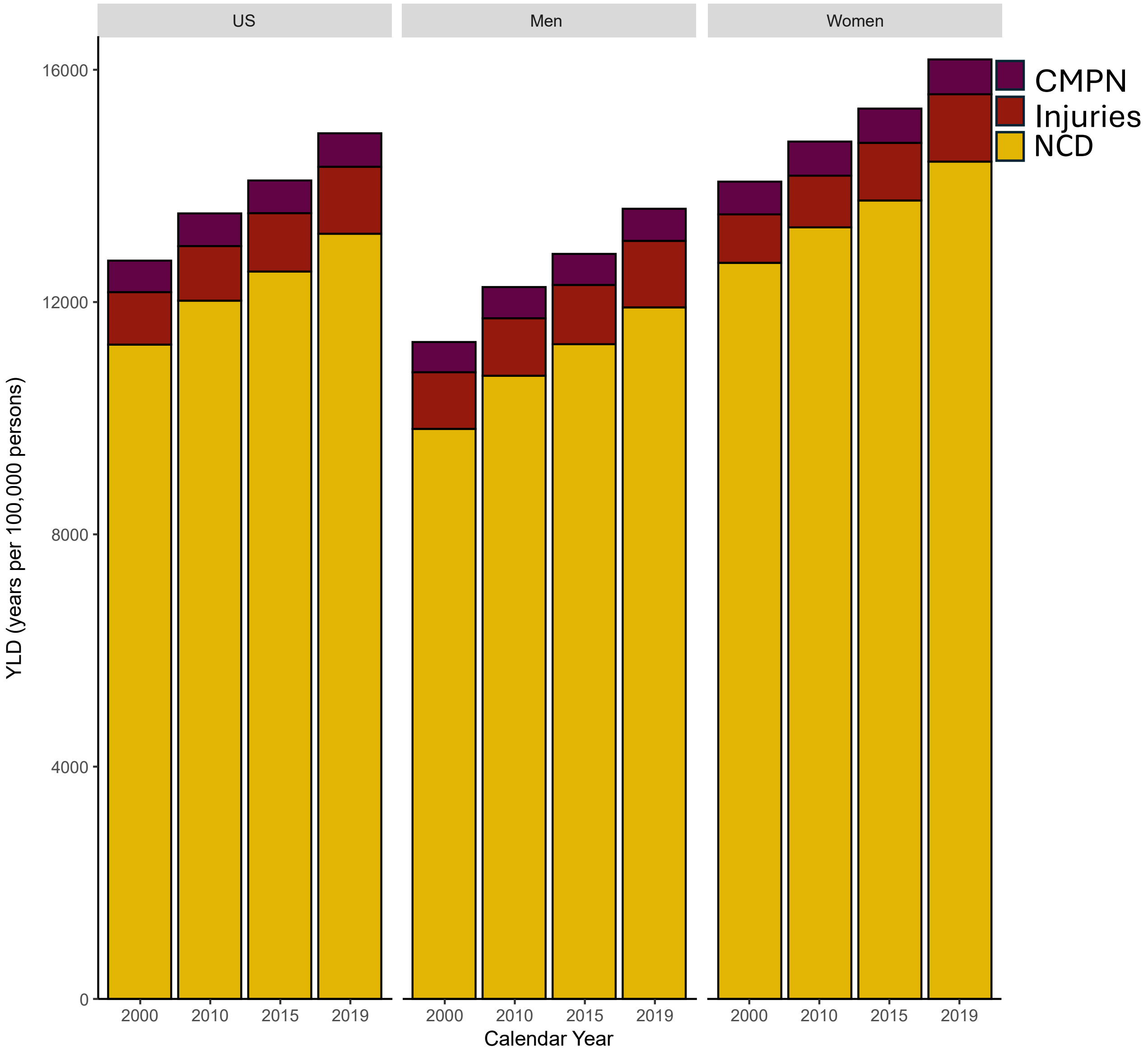

eFigure 4. Years lived with disability (YLD) for noncommunicable diseases (NCD) and injuries, and communicable, maternal, perinatal and nutritional conditions (CMPN) for the United States of America total population, men, and women.

© 2024 Garmany A et al. *JAMA Network Open*.
