# Supplementary material for: Global Healthspan-Lifespan Gaps Among 183 World Health Organization Member States
Source: JAMA Netw Open. 2024 Dec 11;7(12):e2450241. doi: 10.1001/jamanetworkopen.2024.50241 (PMC11635540; doi:10.1001/jamanetworkopen.2024.50241)
Supplement: Supplement 2. — Data Sharing Statement [file jamanetwopen-e2450241-s002.pdf]

Data Sharing Statement

Garmany. Global Healthspan-Lifespan Gaps Among 183 World Health Organization Member States. *JAMA Netw Open*. Published December 11, 2024.
doi:10.1001/jamanetworkopen.2024.50241

Data
Data available: No

Additional Information
Explanation for why data not available: The data are publicly available through the World Health Organization Global Health Observatory.
